# Development and Validation of Differential Diagnosis Models and Nomograms Based on Serum D-Dimer and Other Multimodal Information for Borderline and Benign Epithelial Ovarian Tumors: A Multicenter Study

**DOI:** 10.3390/diagnostics15162035

**Published:** 2025-08-14

**Authors:** Yiqing Zhang, Yayang Duan, Fang He, Chunhua Duan, Junli Wang, Chaoxue Zhang, Yi Zhou

**Affiliations:** 1 Department of Ultrasound, First Affiliated Hospital of Anhui Medical University, Hefei 230022, China; haoyiq2022@126.com (Y.Z.); drduan_yayang@163.com (Y.D.); 15171808560@163.com (F.H.); 18855767637@163.com (C.D.); 2 Department of Ultrasound, Lu’an People’s Hospital of Anhui Province, Lu’an 237000, China; 3 Department of Ultrasound, The Second People’s Hospital, Wuhu 241001, China; wjl980134@163.com

**Keywords:** borderline epithelial ovarian tumors, transvaginal ultrasonography, benign epithelial ovarian tumor, diagnostic model, risk assessment, D-dimer, CA125

## Abstract

**Background:** It is difficult to make a definite diagnosis of borderline epithelial ovarian tumors before surgery. In order to avoid incorrectly classifying tumors as benign, a differential diagnosis model was developed to distinguish between benign and borderline epithelial tumors utilizing multimodal information. **Method:** A multicenter study was conducted. A retrospective analysis of the transvaginal ultrasonography and clinical data of patients who underwent surgery and received pathological diagnoses of borderline and benign epithelial ovarian tumors was conducted. Both Univariate and multivariate logistic regression analyses were used to develop a diagnostic model for borderline epithelial tumors. The efficacy and feasibility of this model were assessed through examination of training, internal validation, and external test sets. **Results:** There was a significant difference in D-dimer levels between borderline and benign epithelial tumors. Abnormal CA125, D-dimer, maximum mass diameter > 10 cm, regular and irregular solid portions, and blood flow in the mass were independent risk factors for borderline epithelial ovarian tumors. The diagnostic model was evaluated by the Hosmer–Lemeshow test and demonstrated strong fitting capabilities. ROC curve analysis of the training set, verification set, and external test set confirmed the model’s predictive ability. **Conclusions**: These independent risk factors may be combined to assess the risk of borderline epithelial ovarian tumors. Our findings will assist novice gynecologic sonographers in distinguishing between benign and borderline epithelial tumors.

## 1. Introduction

Ovarian epithelioid tumor is the most common pathological type of ovarian tumor, which can be divided into benign, malignant, and borderline. Borderline epithelial ovarian tumors (BEOTs) exhibit an intermediate growth pattern and cytological characteristics that lie between those of benign and malignant epithelial tumors. Compared with malignancy, this disease has an earlier age of onset, with a gradual progression, the 5-year and 10-year survival rates are 95% and 90%, respectively [1,2,3,4,5] However, they are prone to invasive implantation in the omentum and other distant sites, leading to frequent relapse and ultimately posing a life-threatening risk [6]. Surgical resection is the recommended course. Consequently, accurate diagnosis and prompt surgical removal are crucial components of effective treatment [7].

The macroscopic characteristics and aggressive behavior of BEOTs are similar to those of both benign and malignant tumors, posing challenges in preoperative diagnosis. Accurate diagnosis often relies on postoperative pathological analysis, necessitating a high level of expertise from pathologists [8,9,10]. Ultrasound has become the imaging modality of choice for routine gynecological examinations, leading to notable enhancements in the diagnostic precision of both benign and malignant ovarian tumors owing to advancements in resolution and the development of various diagnostic models [11,12]. Among the models, O-RADS has the highest sensitivity [13]. Some BEOTs exhibit ultrasonic characteristics that closely resemble those of benign masses, thereby diminishing the efficacy of O-RADS. This limitation contributes to a high rate of incorrect diagnoses of borderline tumors. Hence, this study was undertaken to examine the differential diagnosis between ovarian borderline and benign epithelial tumors.

D-dimers, soluble fibrin degradation products, serve as indirect indicators of thrombotic activity [14]. The latest research indicates that patients with malignant tumors exhibit elevated D-dimer blood levels in comparison to those with benign tumors. D-dimer in the blood of ovarian cancer patients can reach 17 times the normal level. The D-dimer level was normal in benign tumors. D-dimer has been shown to be a good predictor of ovarian cancer [15,16,17,18]. However, the significance of D-dimer in the diagnosis of borderline epithelial ovarian tumors has not been studied. Given the high sensitivity and low specificity of D-dimer, we developed a diagnostic model for distinguishing between ovarian borderline and benign epithelial tumors by integrating sonographic features from vaginal ultrasound and clinical data. This model will assist ultrasound doctors and clinical physicians in quickly completing the process of differential diagnosis during their daily work, thereby avoiding misdiagnosis and delaying the treatment opportunity.

## 2. Materials and Methods

**Figure 1:**
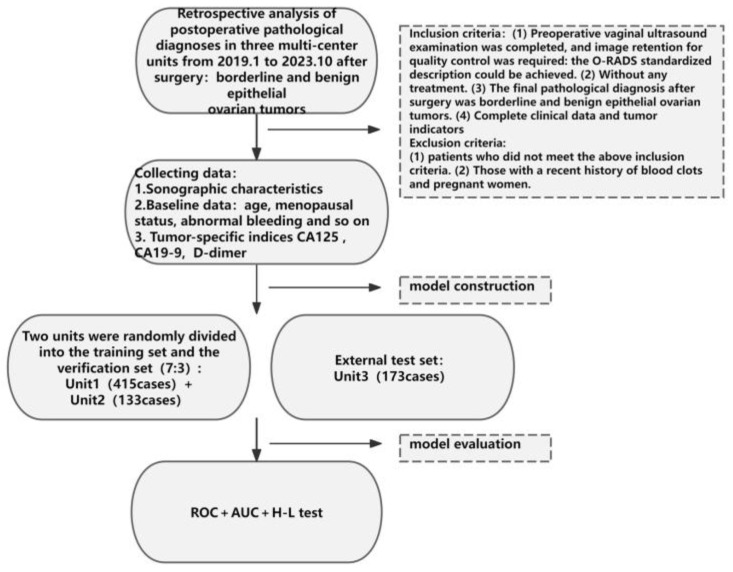
Project flow chart.

We conducted a multicenter study involving three hospitals (Figure 1). The transvaginal ultrasonography and clinical data of patients who underwent surgery at three hospitals between January 2019 and October 2023 and who received pathological diagnoses of borderline and benign epithelial ovarian tumors were retrospectively analyzed. The inclusion criteria were as follows: (1) A transvaginal ultrasound examination should be performed prior to surgery, ensuring the retention of all ultrasound image data, such as grayscale and color energy Doppler data. (2) Without any treatment. (3) The final pathological diagnosis after surgery was borderline and benign epithelial ovarian tumors, including serous tumor, mucinous tumor, endometrioid tumor, clear cell tumor, and transitional cell tumor. Intraoperative frozen sections were not taken as the final result. All pathological results were reviewed by doctors with senior professional titles; (4) Complete clinical data, including age, menopause status, abnormal bleeding, and tumor indicators (CA125 and CA19-9), the most recent preoperative serum D-dimer measurements, were available. The exclusion criteria were as follows: (1) patients who did not meet the above inclusion criteria and (2) those with a recent history of blood clots and pregnant women.

The study was approved by the First Affiliated Hospital of Anhui Medical University’s Institutional Review Board (PJ-2023-07-11). All patients were informed of the procedure and signed informed consent.

### 2.1. Data Collection

Sonographic characteristics: To normalize the descriptions and records of the ultrasonic images, according to the O-RADS [19], when the final pathological results are unknown, the sonographic features of the mass were described and recorded in a standardized manner by two experienced sonographers with over 15 years of ultrasound experience in obstetrics and gynecology. The standardized ultrasound terms for ovarian tumors made in O-RADS were recorded, e.g., the maximum length of the mass and determining whether there was a solid part, solid part shape, single or multilocular structure, whether there was a blood supply inside the mass, and whether there was ascites.

To reduce subjectivity in ultrasound feature description among different observers, a random sample of 30 cases underwent Kappa agreement testing. The kappa values for the description of each ultrasound feature among observers were all greater than 0.75, indicating strong inter-observer agreement. If the feature description is unclear, it will be reviewed and defined by a senior obstetric ultrasound physician.

Baseline data, including variables such as age, menopausal status, and the presence of abnormal bleeding, pathology, were gathered.

The aim of this study was to investigate the differential diagnosis between ovarian borderline and benign epithelial tumors. To achieve this goal, the tumor-specific indices CA125 and CA19-9, as well as serum D-dimer indicators, were collected to assess their value in the diagnostic process. Due to the different detection methods and reagents, the reference standards for each indicator are also different. Secondly, some variables are influenced by age. The reference standards vary among different age groups. Based on the reference values established by the detection kit, we classified the results of each variable as normal or abnormal. Unit1: CA125 (0–35 U/mL), CA19-9 (0–37 U/mL), D-dimer (<65 Y:<0.55 mg/L; ≥65 Y: <0.8 mg/L); Unit1: CA125 (0–30.2 U/mL), CA19-9 (0–37 U/mL), D-dimer (<65 Y: <0.55 mg/L; ≥65 Y: <0.8 mg/L); Unit3: CA125 (0–35 U/mL), CA19-9 (0–37 U/mL), D-dimer (<65 Y: <0.50 mg/L; ≥65 Y: <0.8 mg/L)

### 2.2. Statistical Analysis

Guided by the principle of simple randomization, the original datasets from two independent sources were partitioned into a 70% training set and a 30% validation set through multiple iterations of independent random sampling. Measurement data that deviate from a normal distribution are denoted by M (*P*_25_, *P*_75_), whereas count data are represented by cases and percentages. The rank sum test and chi-square test were employed to assess the clinical characteristics of patients in both the training and validation sets and to compare the clinical features of patients with benign and borderline tumors within the training set. Univariate logistic regression was employed to examine the variables influencing borderline ovarian epithelial tumors. Variables with a significance level of *p* < 0.05 in the Univariate logistic regression analysis were subsequently incorporated into the multivariate logistic regression analysis to delve deeper into the factors impacting borderline ovarian epithelial tumors. On the basis of the findings of a multivariate logistic regression analysis, a prediction model was developed via a nomogram, and a calibration curve was constructed. The goodness of fit of the model was assessed via the Hosmer-Lemeshow (H-L) test. A receiver operating characteristic (ROC) curve was generated, and the area under the curve (AUC) was employed as a metric for assessing the model’s ability to differentiate between borderline tumors. An additional multicenter participating unit was subsequently incorporated into the model as an external test set. A ROC curve was constructed, and the H–L test was used to validate the model. All the statistical analyses were conducted via R version 4.2.3, with statistical significance defined as *p* < 0.05.

## 3. Results

A total of 548 patients (Unit1 + Unit2), comprising 378 with benign epithelial tumors and 170 with borderline epithelial tumors, with an average age of 37 years, were randomly allocated into training and validation sets at a ratio of 7:3, resulting in 383 patients in the training set and 165 in the validation set. No statistically significant disparity was observed between the training and validation sets (all *p* > 0.05) (Table 1).

The age of patients with BEOTs, the proportion of menopausal patients, the proportion of CA125, CA19-9, and D-dimer abnormalities, the proportion of masses with a maximum length >10 cm, the proportion of regular and irregular solid parts, and the proportion of blood supply in the tumor were greater than those of patients with benign epithelial tumors (all *p* < 0.05) (Table 2).

The findings of the univariate logistic regression analysis indicate that age, menopausal status, abnormal CA125, CA19-9, and D-dimer, a tumor size exceeding 10 cm; and the presence of irregular shapes, regular shapes, and blood supply within the mass were statistically significant risk factors for borderline tumors (all *p* < 0.05). Multivariate logistic regression analysis revealed that abnormal CA125 and D-dimer levels, a maximum mass diameter exceeding 10 cm, the presence of regular and irregular solid portions, and the presence of blood flow within the mass were identified as independent risk factors for borderline tumors (all *p* < 0.05). The *OR* values were 2.44 (95% CI: 1.07~5.56), 20.26 (95% CI: 1.07~5.56), 3.44 (95% CI: 1.49~7.92), 9.47 (95% CI: 4.10~21.89), 175.96 (95% CI: 16.00~1 934.89) and 3.33 (95% CI: 1.05~10.54) (all *p* < 0.05) (Table 3).

At the same time, in order to avoid the distortion of the model results caused by the multicollinearity problem, we performed the variance expansion factor (VIF) test, and the results showed that the VIF values were <5 and there was no multicollinearity (Figure S1).

**Figure 2:**
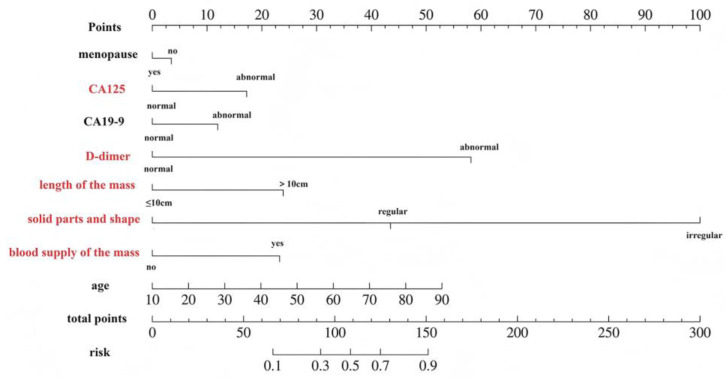
A nomogram based on risk factors. Red highlights indicate independent risk factors, which are used for model scoring.

The nomogram model for borderline epithelial tumors, constructed via multivariate logistic regression analysis, is depicted in Figure 2. The individual scores corresponding to various values of each predictor are represented at the top of the column chart, whereas the cumulative scores at the bottom reflect the total scores derived from all the predictors. For the application of this nomogram diagnostic model, if a 43-year-old premenopausal woman has no abnormal bleeding, CA125 and D-dimer levels are increased. A mass > 10 cm in size was found in the pelvic cavity, with solid components and an irregular shape, and the solid part was detected and the blood supply was determined. According to the model, the CA125 and D-dimer levels were increased to 75 points, the maximum diameter was >10 cm, and the solid blood supply was 150 points. When the total score was 220, the mass had a 90% + risk of being borderline in a BEOTs.

**Figure 3:**
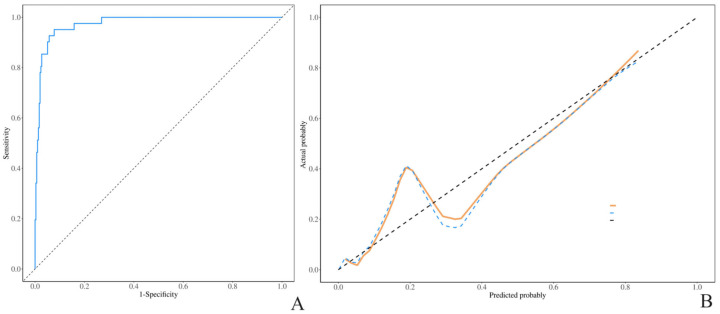
(**A**,**B**): ROC curve and H- correction curve of the training set.

**Figure 4:**
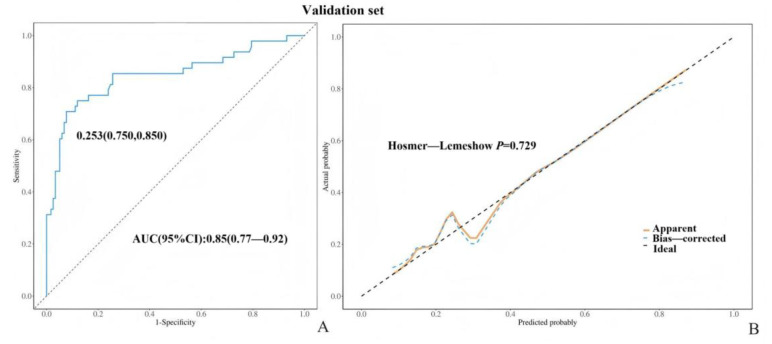
(**A**,**B**): ROC curve and H-L correction curve of the validation set.

**Figure 5:**
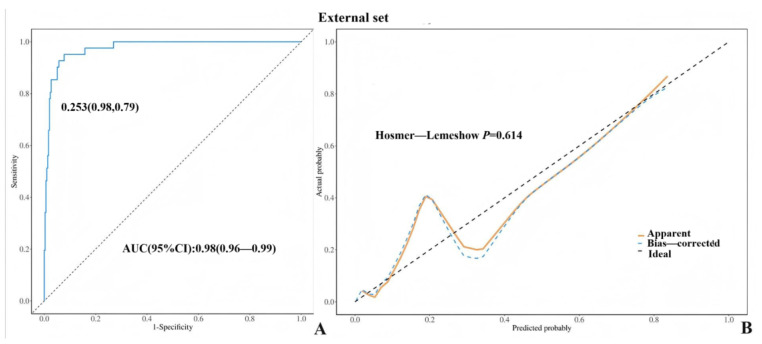
(**A**,**B**): ROC curve and H-L correction curve of the external test set.

The ROC curve was constructed on the basis of the classification of tumors as borderline. The AUC values for both the training and validation sets exceeded 0.80, with values of 0.95 (95% confidence interval: 0.93–0.97) and 0.85 (95% confidence interval: 0.77–0.92), respectively. According to the ROC curve, the maximum entry index of the training set was 0.253, the sensitivity was 0.870, and the specificity was 0.910, indicating the high prediction accuracy of the model (Figure 3A,B and Figure 4A,B). The external test set (Unit3) is introduced into the prediction model (BEOTs: 41 cases; Benign: 132 cases), and the results show that the AUC value of the external test set is 0.98 (95% CI: 0.96~0.99) (Figure 5A,B), indicating that the prediction model established in this study has good prediction ability. Additionally, we externally validated the model using the subset of patients aged ≥50 years from the validation cohort, sensitivity: 0.94, further confirming the model’s robust discriminatory performance. The H–L test results revealed that the training set and verification set models had good fitting ability (*p* > 0.05), indicating that the model had good predictive performance.

**Figure 6:**
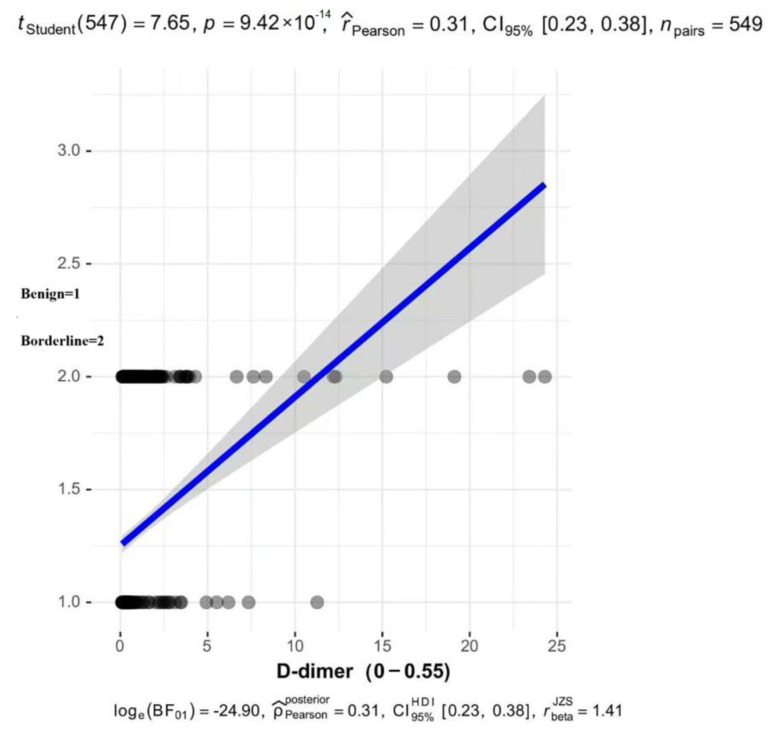
Correlation Analysis of D-dimer and BEOTs. The abscissa and ordinate are two variable values, and the blue line is the optimal fit line found according to the ”least squares” criterion of all the straight lines that pass through these scatters, it makes the sum of the squares of the vertical distance (residual) of all data points to the line the smallest.

To better verify the correlation between D-dimer and borderline tumors, we conducted linear regression on the D-dimer measurements of Unit1 and Unit2 with exactly the same reference values. The results showed that D-dimer in serum was positively correlated with borderline tumors, with r = 0.31. According to Cohen’s criteria, there was a moderate correlation (Figure 6).

## 4. Discussion

Based on the characteristics of ultrasound O-RADS, clinical data, and laboratory indicators, we constructed a model capable of rapidly differentiating BEOTs from benign tumors. It was evaluated through methods such as a validation set, an external test, and an H-L test to verify the predictive efficacy of the model. This will help ultrasound doctors and clinicians make rapid differential diagnoses before surgery and avoid misdiagnosing BEOTs as benign tumors.

BEOTs are distinguished by having an atypical composition exceeding 10%, displaying certain morphological features commonly observed in malignant tumors but lacking destructive interstitial infiltration [20]. The age of onset for this condition precedes that of malignant tumors, with a slow growth rate and nonspecific clinical manifestations. Approximately 30% of patients are asymptomatic, with pelvic or abdominal masses only being detected through physical examination or routine gynecologic ultrasound [8]. While borderline tumors exhibit a lower degree of malignancy than malignant tumors do, they present malignant characteristics such as infiltration and metastasis, lymph node invasion, a high likelihood of recurrence, and the potential for life-threatening consequences. Surgical resection is the preferred treatment, with early diagnosis playing a pivotal role in successful treatment outcomes. However, compared with benign and malignant tumors, borderline ovarian tumors have overlapping signs, making their preoperative diagnosis difficult. If it is misdiagnosed as a benign tumor, it will delay the best treatment opportunity for the patient. From the perspective of formulating clinical treatment plans, the differentiation of diagnosis between benign and borderline tumors is given more importance [21,22]. Whereas epithelial tumors are the most common type of ovarian tumors [23], this study aims to increase the detection rate of BEOTs by investigating the utility of clinical diagnostic models in distinguishing between borderline and benign epithelial tumors, thereby preventing delays in optimal surgical intervention.

In the previous reported research, Research on ovarian tumors has focused primarily on developing models for distinguishing between benign and malignant tumors, with limited attention given to borderline tumors, particularly in terms of predicting the risk of borderline tumors compared with that of benign tumors [24,25]. To increase the precision and reliability of our model, we conducted a multicenter study that integrated various forms of data, including clinical information and sonogram characteristics. We selected variables previously reported in the literature to be associated with borderline ovarian tumors (BOTs), including: age, menopausal status, presence of abnormal vaginal bleeding, CA125, and CA19-9. In addition, we included D-dimer for the first time in this context [26,27,28]. Malignant tumor cells can release procoagulant substances, induce platelet activation, and cause endothelial injury, thereby promoting the conversion of fibrinogen into fibrin and the formation of microthrombi. These processes trigger compensatory activation of the fibrinolytic system, leading to increased D-dimer production. Meanwhile, tumor cells secrete urokinase-type plasminogen activator (uPA), which degrades fibrin to generate D-dimer, while simultaneously promoting the release of matrix metalloproteinases (MMPs). These enzymes disrupt the basement membrane, thereby facilitating tumor cell invasion [29,30]. Studies have demonstrated the utility of D-dimer in the prediction of ovarian malignancy. Additional studies have suggested that D-dimer levels may also have prognostic value in predicting metastasis, progression, and mortality in various types of cancer [14,15,31]. Therefore, combining D-dimer with other tumor markers may improve the diagnostic efficiency for malignancies. Borderline tumors exhibit biological characteristics intermediate between malignant and benign neoplasms. The diagnostic and differential diagnostic value of D-dimer in these tumors remains unclear. Our findings revealed a statistically significant increase in D-dimer levels in borderline epithelial ovarian tumors compared with those in benign tumors. The correlation analysis demonstrated a statistically significant but moderate positive association between D-dimer levels and borderline ovarian tumors (Pearson’s r = 0.31, *p* < 0.05). Other factors may significantly influence borderline tumors, making them insufficient for a standalone clinical diagnosis. Therefore, we incorporated these factors into our model study, necessitating a combined assessment with additional biomarkers for comprehensive diagnostic evaluation.

These findings may lead to new ideas for the early differential diagnosis of other tumors. For ultrasound images, we combined grayscale images and color images from a conventional vaginal ultrasound. To increase the precision of the imaging data, we assessed the mass’s actual blood supply via color energy Doppler, which is less susceptible to variations in instrument parameters than traditional color Doppler techniques are. Additionally, senior gynecologist sonographers described and recorded image features according to the standardized O-RADS criteria. We evaluated the maximum length diameter of the tumor, whether it had solid components, whether the solid part was regular in shape, whether it had a single-locular or multilocular structure, and whether there was a blood supply inside the tumor. Considering the value of the actual blood supply to the evaluation of the tumor, we did not include the blood supply assessment at the edge or around the tumor. The results of the multimodal analysis of the included clinical data, laboratory parameters, imaging features, and other variables revealed that abnormal CA125 and D-dimer levels, a maximum diameter of the mass > 10 cm, a regular and irregular solid portion, and blood flow in the mass were identified as independent risk factors for BEOTs. The evaluation of the model we constructed also confirmed that it has strong predictive capabilities. These findings will help differentiate between benign and borderline cases, and support inexperienced gynecological ultrasound practitioners in their daily clinical practice.

Our research also has some limitations: Although this is a multicenter study, the sample size remains relatively small due to the low incidence of borderline tumors, which may limit the study’s ability to detect subtle differences. As a retrospective study, our analysis was constrained by the limited availability of certain ovarian tumor-related biomarkers reported in the literature. Notably, key variables such as HE4 and AFP were systematically missing from the dataset and could not be incorporated into the final model. Second, there might be slight differences in the detection reagents and methods among the multi-center units, and the measured values and reference ranges were also different. Therefore, in the data processing of this study, it was only classified as normal and abnormal according to the reference value ranges of the laboratories of each unit. In the later prospective studies, we will unify the measurement methods and reagents of each unit and explore the relationship between serological indicators and borderline tumors. In summary, the limited sample size and data constraints have resulted in excessively wide confidence intervals for certain parameters. Although we have evaluated and validated the model through multiple approaches, further efforts are needed to improve its performance and precision. In future prospective studies, we will expand the sample size, standardize data homogenization, optimize data processing methods, and explore its clinical value in borderline tumors.

## 5. Conclusions

In conclusion, our findings indicate a significant difference in D-dimer levels between borderline and benign epithelial ovarian tumors; abnormal levels of serum CA125 and D-dimer, a maximum mass diameter exceeding 10 cm, the presence of regular and irregular solid portions, and the detection of blood flow within the mass are identified as independent risk factors for borderline tumors. On this basis, the model we constructed is conducive to the differential diagnosis of borderline and benign epithelial ovarian tumors, and it should be especially helpful for less experienced sonographers to avoid classifying borderline tumors as benign, which delays surgery past the optimal time.

## Figures and Tables

**Table 1:** Comparative analysis of training set and verification set features [*M* (*P*_25_, *P*_75_), *n* (%)].

Variate	Total (*n* = 548)	Validation Set (*n* = 165)	Training Set (*n* = 383)	*Z*/*χ*^2^	*p*
age	37.00 (29.00, 49.00)	35.00 (27.00, 49.00)	37.00 (30.00, 49.00)	−1.67	0.096
pathology				0.41	0.521
benign	378 (68.98)	117 (70.91)	261 (68.15)		
borderline	170 (31.02)	48 (29.09)	122 (31.85)		
menopause				0.26	0.611
no	448 (81.75)	137 (83.03)	311 (81.20)		
yes	100 (18.25)	28 (16.97)	72 (18.80)		
abnormal bleeding				0.08	0.784
no	542 (98.91)	164 (99.39)	378 (98.69)		
yes	6 (1.09)	1 (0.61)	5 (1.31)		
CA125				1.68	0.194
normal	326 (59.49)	105 (63.64)	221 (57.70)		
abnormal	222 (40.51)	60 (36.36)	162 (42.30)		
CA19-9				0.01	0.903
normal	417 (76.09)	125 (75.76)	292 (76.24)		
abnormal	131 (23.91)	40 (24.24)	91 (23.76)		
D-dimer				0.83	0.361
normal	370 (67.52)	116 (70.30)	254 (66.32)		
abnormal	178 (32.48)	49 (29.70)	129 (33.68)		
maximum length diameter of the mass (cm)				0.08	0.777
≤10	403 (73.54)	120 (72.73)	283 (73.89)		
>10	145 (26.46)	45 (27.27)	100 (26.11)		
solid parts and forms				0.63	0.731
no	321 (58.58)	95 (57.58)	226 (59.01)		
regular	185 (33.76)	59 (35.75)	126 (32.90)		
irregular	42 (7.66)	11 (6.67)	31 (8.09)		
single or multilocular				0.35	0.553
single	253 (46.17)	73 (44.24)	180 (47.00)		
multiple	295 (53.83)	92 (55.76)	203 (53.00)		
internal blood supply of the mass				0.67	0.414
no	475 (86.68)	146 (88.48)	329 (85.90)		
yes	73 (13.32)	19 (11.52)	54 (14.10)		
ascites				2.95	0.086
no	524 (95.62)	154 (93.33)	370 (96.61)		
yes	24 (4.38)	11 (6.67)	13 (3.39)		

Z: Mann–Whitney; *χ*^2^: chi-square test.

**Table 2:** Comparison of clinical features of benign and borderline epithelial ovarian tumors in the training set [M (P_25_, P_75_), *n* (%)].

Variate	Total (*n* = 383)	Benign (*n* = 261)	Borderline (*n* = 122)	Z/*χ*^2^	*p*
age	37.00 (30.00, 49.00)	36.00 (29.00, 47.00)	44.00 (33.00, 53.75)	−3.58	<0.001
menopause				5.13	0.024
no	311 (81.20)	220 (84.29)	91 (74.59)		
yes	72 (18.80)	41 (15.71)	31 (25.41)		
abnormal bleeding				3.40	0.065
no	378 (98.69)	260 (99.62)	118 (96.72)		
yes	5 (1.31)	1 (0.38)	4 (3.28)		
CA125				36.99	<0.001
normal	221 (57.70)	178 (68.20)	43 (35.25)		
abnormal	162 (42.30)	83 (31.80)	79 (64.75)		
CA19-9				14.97	<0.001
normal	292 (76.24)	214 (81.99)	78 (63.93)		
abnormal	91 (23.76)	47 (18.01)	44 (36.07)		
D-dimer				168.32	<0.001
normal	254 (66.32)	229 (87.74)	25 (20.49)		
abnormal	129 (33.68)	32 (12.26)	97 (79.51)		
maximum length diameter of the mass (cm)				33.40	<0.001
≤10	283 (73.89)	216 (82.76)	67 (54.92)		
>10	100 (26.11)	45 (17.24)	55 (45.08)		
solid parts and forms				110.35	<0.001
no	226 (59.01)	195 (74.71)	31 (25.41)		
regular	126 (32.90)	65 (24.90)	61 (50.00)		
irregular	31 (8.09)	1 (0.37)	30 (24.59)		
single or multilocular				0.09	0.769
single	180 (47.00)	124 (47.51)	56 (45.90)		
multilocular	203 (53.00)	137 (52.49)	66 (54.10)		
internal blood supply of the mass				76.75	<0.001
no	329 (85.90)	252 (96.55)	77 (63.11)		
yes	54 (14.10)	9 (3.45)	45 (36.89)		
ascites				2.04	0.153
no	370 (96.61)	255 (97.70)	115 (94.26)		
yes	13 (3.39)	6 (2.30)	7 (5.74)		

Z: Mann–Whitney; *χ*^2^: chi-square test.

**Table 3:** Logistic regression analysis results.

Variate	Univariate Logistic Regression		Multivariate Logistic Regression	
	OR (95% CI)	*p*	OR (95% CI)	*p*
age	1.03 (1.01~1.04)	<0.001	1.03 (0.99~1.08)	0.090
menopause				
no	1.00 (Reference)		1.00 (Reference)	
yes	1.83 (1.08~3.09)	0.025	0.84 (0.21~3.37)	0.801
abnormal bleeding				
no	1.00 (Reference)			
yes	8.81 (0.97~79.71)	0.053		
CA125				
normal	1.00 (Reference)		1.00 (Reference)	
abnormal	3.94 (2.50~6.20)	<0.001	2.44 (1.07~5.56)	0.035
CA19-9				
normal	1.00 (Reference)		1.00 (Reference)	
abnormal	2.57 (1.58~4.18)	<0.001	1.85 (0.82~4.20)	0.139
D-dimer				
normal	1.00 (Reference)		1.00 (Reference)	
abnormal	27.77 (15.63~49.32)	<0.001	20.26 (9.19~44.68)	<.001
maximum length diameter of the mass (cm)				
≤10	1.00 (Reference)		1.00 (Reference)	
>10	3.94 (2.44~6.37)	<0.001	3.44 (1.49~7.92)	0.004
solid parts and forms				
no	1.00 (Reference)		1.00 (Reference)	
regular	5.90 (3.53~9.88)	<0.001	9.47 (4.10~21.89)	<.001
irregular	188.71 (24.83~1434.10)	<0.001	175.96 (16.00~1934.89)	<.001
single room or multiple rooms				
single room	1.00 (Reference)			
multiple room	1.07 (0.69~1.64)	0.769		
internal blood supply of the mass				
no	1.00 (Reference)		1.00 (Reference)	
yes	16.36 (7.65~34.98)	<0.001	3.33 (1.05~10.54)	0.041
ascites				
no	1.00 (Reference)			
yes	2.59 (0.85~7.87)	0.094		

## Data Availability

The datasets used and/or analyzed during the current study are available from the corresponding author on reasonable request.

## Supplementary Materials

The following supporting information can be downloaded at https://www.mdpi.com/article/10.3390/diagnostics15162035/s1, Figure S1: Variance inflation factor.
