# Supplementary figures and images for: Development and Validation of Differential Diagnosis Models and Nomograms Based on Serum D-Dimer and Other Multimodal Information for Borderline and Benign Epithelial Ovarian Tumors: A Multicenter Study
Source: Diagnostics (Basel). 2025 Aug 14;15(16):2035. doi: 10.3390/diagnostics15162035 (PMC12385617; doi:10.3390/diagnostics15162035)

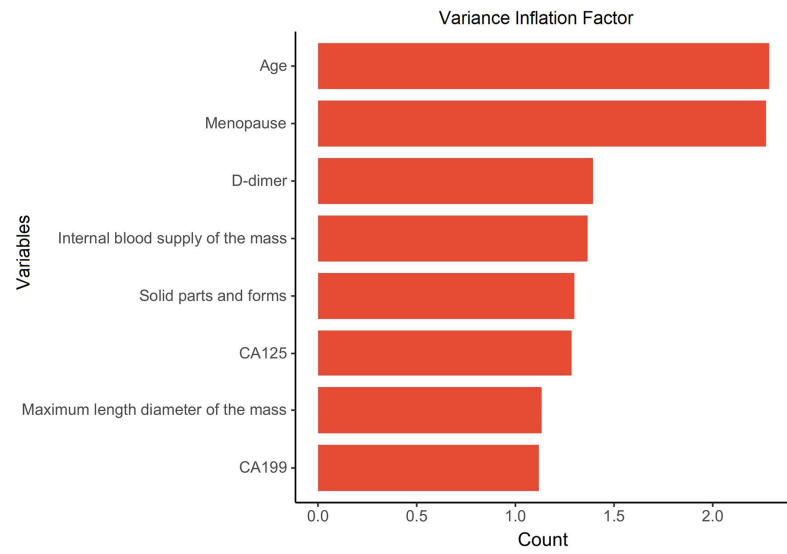

Figure S1: Variance Inflation Factor.

Supplement: Supplementary file 1 [file diagnostics-15-02035-s001.zip › Figure S1.pdf]
